# The Trueness between Conventional Impression and Different Intraoral Scanners for All-on-4 Implants: An *In vitro* Comparative Study

**DOI:** 10.1055/s-0045-1811961

**Published:** 2025-10-09

**Authors:** Osamah A. Alsulimani, Abdulrahman J. Alhaddad, Samar H. Abuzinadah, Saeed J. Alzahrani, Hamed S. Alghamdi, Farah A. Ghander, Refad M. Magadmi

**Affiliations:** 1 Department of Oral Diagnostic Sciences, Faculty of Dentistry, King Abdulaziz University, Jeddah, Saudi Arabia; 2 Department of Oral and Maxillofacial Prosthodontics, Faculty of Dentistry, King Abdulaziz University, Jeddah, Saudi Arabia; 3 Department of Restorative Dentistry, Faculty of Dentistry, King Abdulaziz University, Jeddah, Saudi Arabia; 4 Department of Oral and Maxillofacial Surgery and Diagnostic Science, Faculty of Dental Medicine, Umm Al-Qura University, Makkah, Saudi Arabia; 5 Internship Program, Faculty of Dentistry, King Abdulaziz University, Jeddah, Saudi Arabia

**Keywords:** degree of deviation, All-on-4, trueness, intraoral scan, precision, accuracy

## Abstract

**Objectives:**

To assess and compare the trueness (dimensional discrepancy and degree of deviation) of various methods of impressions for All-on-4 implants.

**Materials and Methods:**

This investigation employed a single-piece artificial mandibular jaw with four implants arranged in an All-on-4 configuration. Three impression methods were compared: one open-tray conventional impression digitized after pouring, and two intraoral scanners, TRIOS 5 and Runyes 3DS 3.0. A reference scan (control) was conducted with a laboratory-based scanner. All scans were performed using scan bodies and exported as Standard Tessellation Language (STL) files. A total of 30 STL scans were produced ( *n* = 10). The dimensional discrepancy (along the *X* , *Y* , and *Z* axes) and the overall degree of deviation in the position were assessed. Data analysis was conducted using Brown–Forsythe one-way analysis of variance and Tamhane's post hoc tests ( *p* < 0.05).

**Results:**

The mean degree of deviation for scan bodies was as follows: TRIOS 5 (1.11 ± 0.06 mm), Runyes 3DS (1.02 ± 0.05 mm), and conventional (0.82 ± 0.16 mm). Statistically significant differences were found among all impression methods ( *p* < 0.05). While the conventional method showed the highest trueness, it had the greatest standard deviation (SD, 0.16), which was the least consistent among them. The Runyes 3DS scans displayed the highest precision with the degree of deviation of 0.05 (± SD). Dimensional discrepancies mainly occur on the *Z* -axis across all three impression methods. Conventional impressions showed statistically significant discrepancies in the *Y* - and *Z* -axes, while TRIOS 5 images had statistically significant discrepancies in the *X* - and *Z* -axes. Runyes 3DS readings were statistically significantly discrepant in the *Z* -axis.

**Conclusion:**

While both conventional methods and digital scans have their merits, traditional impression methods may offer improved trueness in full-arch implant cases. Utilizing the open-tray system with suitable materials and methods can enhance precision and lead to more reliable outcomes.

## Introduction

Implant impression represents a critical step in the fabrication of an implant-supported fixed dental prosthesis. This procedure necessitates the accurate transmission of the implants' position and angle within the mouth, which is essential for achieving optimal treatment outcomes. 1 2 Impression making can be executed through two primary methods: the conventional method, which employs elastomeric impression materials to record implant positions via a physical cast, and the digital approach, which utilizes optical scanning technologies, including intraoral scanners (IOSs) and laboratory-based scanners (LBSs). Regardless of the chosen method, the objective remains to accurately transfer the intraoral position of dental implants to either a physical working cast or a digital virtual model. 2

IOSs are sophisticated dental devices designed for capturing direct optical impressions. They consist of a handheld camera (hardware), a computer, and specialized software. 3 These advanced devices utilize three-dimensional (3D) in-motion video imaging technology alongside measurement systems to capture the shape and size of dental arches, thereby reproducing 3D models of the teeth, soft tissues of the oral cavity, and implant scan bodies. 4 The advent of such technologies has significantly transformed the field of dentistry, particularly in the domains of prosthodontics and implantology, by markedly reducing scanning time when compared with conventional impression methods, eliminating the need for physical cast storage, and minimizing patient discomfort during the impression-making process. 5

While conventional impressions have long been regarded as the gold standard in clinical dentistry, their usage has been accompanied by several challenges, including the necessity for material preparation, susceptibility to impression distortion, high sensitivity to technique, protracted clinical workflows, and increased patient discomfort. 6 7 Conversely, digital impressions present distinct advantages such as enhanced efficiency, precision, and seamless integration with computer-aided design and manufacturing (CAD/CAM) systems. 7 The IOS technology has found successful applications across various dental situations, including fixed partial dentures, complete dentures, maxillofacial prostheses, and implant dentistry. 5 8 In the context of implant-supported prosthetics, IOSs have improved processes spanning from treatment planning to the fabrication of definitive prostheses. 9 This evolution has not only transformed the daily routines of both dental practitioners and technicians, but has also facilitated a more streamlined and precise approach to impression making.

Presently, a diverse array of scanners is available on the market, employing various optical technologies, acquisition methods, and reconstruction algorithms. 10 11 12 13 IOSs predominantly utilize laser beam or structured light technology, 4 5 while LBSs incorporate three main technologies: laser beam, structured light, and contact-based methods. 14 Over the last 10 years, there has been a significant rise in the accessibility of optical IOSs, each based on different technologies that may influence the quality of clinical outcomes. 15 16

The exact positioning of dental implants is essential for the success and durability of prostheses supported by implants. 17 18 The positioning of implants has a significant impact on biomechanical stability, esthetic outcomes, and functional efficacy; thus, the accuracy of the impression process is of utmost importance. 19 Within the domain of implant dentistry, the impression technique is essential for effectively transferring the intraoral position and angular orientation of dental implants onto the gypsum cast, which plays a vital role in the success of implant-borne full-arch fixed prostheses, such as the All-on-4 therapeutic approach. 19 Achieving a passive fit of the prosthesis is crucial in full-arch cases 20 and is integral to preventing complications such as mechanical stress and eventual implant failure. 10 While the literature reports that the acceptable range for passive fit varies between 10 and 150 µm, it is recommended that this range be confined to between 30 and 50 µm to mitigate the risk of mechanical and biological complications. 21 22

The process of obtaining optical impressions for All-on-4 edentulous arches poses considerable challenges, particularly when dealing with tilted implants. 23 The lack of reference points (teeth) in completely toothless jaws makes the stitching process challenging. Additionally, the presence of movable tissues and saliva, combined with the pink hue of the soft tissue, further complicates full-arch imaging. 23 Additionally, factors such as the dimensions, colors, and design of the scanning body, in conjunction with the positioning and accessibility of the implants, significantly affect the accuracy of full-arch implant scanning. 24 Furthermore, the employment of various IOSs appears to have a substantial impact on the fidelity of implant position documentation. 25

Accuracy is defined as the “closeness of agreement between a measured quantity value and a true quantity value of a measurand.” 25 26 This concept encompasses two primary components: trueness and precision, which, while distinct, are complementary. Trueness pertains to the ability of a measurement to closely align with the actual value of the quantity being assessed, whereas precision reflects the consistency or repeatability of measurements taken from the same object. 10 For an IOS to function effectively, it must be capable of accurately capturing all intricate details of the scan, producing a virtual 3D model that closely resembles the reference model with minimal deviations. This is critical to ensure that the digital impressions generated by the IOS are reliable and suitable for clinical applications.

In the present study, the authors prioritize trueness over precision, focusing on evaluating the degree to which the IOSs can replicate the actual geometry of the scanned object. The emphasis on trueness is warranted because it directly impacts clinical outcomes, ensuring that digital impressions accurately reflect actual anatomical structures' dimensions, which are vital for effective treatment planning, appropriate prosthesis fit, and the minimization of errors that could result in complications. 27 While precision guarantees consistency in repeated measurements, it does not necessarily ensure accuracy in reflecting true anatomical values; therefore, trueness is the more pertinent metric for assessing the performance of IOS technology in this research.

Despite the critical importance of these aspects, limited studies have investigated the accuracy of contemporary IOSs. Lots of existing research focuses on first-generation devices and has not evaluated their performance in All-on-4 implant scenarios, highlighting a significant gap in understanding the capabilities of newer IOSs. This study aims to address this gap by conducting an *in vitro* investigation to assess the impact of various IOSs on the trueness of scanning in All-on-4 implants situated in a completely edentulous mandible. The null hypotheses (H _0_ ) formulated for this study postulate that: (1). there exists no statistically significant difference in the degree of deviation of scan bodies' position for All-on-4 implants between the various methods of impressions and (2) there is no statistically significant difference in the dimensional discrepancy of the scan body zone for All-on-4 implants between the various methods of impressions.

## Materials and Methods

### Model Preparation

A total of four SuperLine implants (4.5 × 10 mm; Dentium, South Korea) were placed in an All-on-4 configuration by an experienced periodontist, complying with the manufacturer's guidelines. The setup included two implants placed vertically in the canine region, and two implants angulated distally in the second premolar area, all embedded in a one-piece artificial jaw model (EasyinSmile, United States).

### Grouping

The study involved two IOSs: the TRIOS 5 (3Shape, Copenhagen K, Denmark), designated as T, and the Runyes 3DS 3.0 (Runyes, Zhejiang, China), referred to as R. Additionally, a conventional silicone impression method was utilized, denoted as C. In total, 30 impressions were obtained, with *n* = 10 for each method.

### Impression Taking

**Fig. 1:**
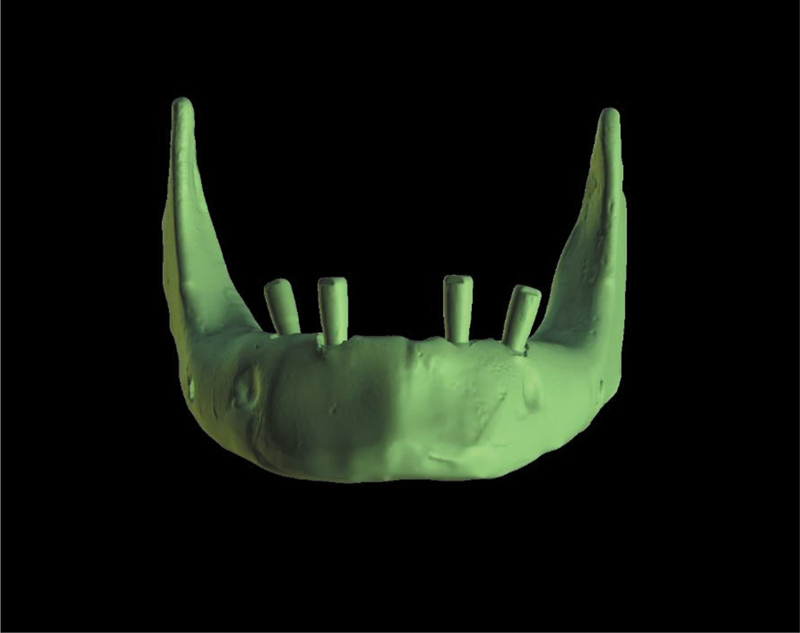
Reference STL scan.

An initial reference scan was conducted on the artificial jaw using an LBS (KaVo ARCTICA AutoScan, Kavo Dental, Germany), incorporating four scan bodies (4.0 × 10 mm, SuperLine, Dentium, South Korea) securely attached to the implants ( Fig. 1 ). All scan bodies were hand-tightened initially and then secured to the implants using a calibrated torque wrench (Dentium) set to the manufacturer's recommended 15 Ncm. Table 1 summarizes the properties of the scanners used in this study. This scanning procedure was executed by a certified technician in accordance with the manufacturer's protocols, and the resultant data were exported in Standard Tessellation Language (STL) format. After the reference scan, the scan bodies were left undisturbed to facilitate subsequent impressions utilizing the two IOSs. Calibration of both scanning devices was performed to ensure precision, followed by a standardized scanning protocol involving a one-way sweep (buccal, occlusal, and lingual surfaces). A scan was deemed complete when it thoroughly encompassed all significant features of the master model without notable gaps and captured the entirety of the scan bodies. This process was meticulously replicated 10 times for each scanner type, strictly adhering to the manufacturer's specifications, with output in STL format for further analysis.

**Table 1:** Properties of the scanners used in the study

Scanner	Runyes 3DS 3.0	TRIOS 5	KaVo ARCTICA AutoScan
Manufacturer	Runyes, Zhejiang, China	3Shape, Copenhagen K, Denmark	Kavo Dental, Biberach, Germany
Imaging principle	Optical continuous video collection	Blue laser and structured light technology	Structured light and laser triangulation
Light source	LED light source without radiation	LED as a light source	Striped light projection
Scan speed	90 s	Captures 2,400 images per second	1 min for a single stump as framework 3 min for a three-unit bridge
Accuracy	<20 μm	100 μm	20 μm

Abbreviation: LED, light-emitting diode.

In the conventional impression protocol, the scan bodies were extracted from the implants, and open-tray impression copings (4.0 × 15 mm, SuperLine, Dentium) were installed and hand-tightened at the implant level. The impression copings were then fastened using a calibrated torque wrench (Dentium) adjusted to the manufacturer's suggested setting of 15 Ncm. Despite existing literature recommending the open-tray splinting technique and abutment level impression for angled implants to enhance full-arch accuracy, this approach was deliberately not utilized. The intention was to replicate the digital scanning conditions and to precisely identify the shortcomings of the silicone impression technique without incorporating additional measures that could mitigate these imperfections. A custom open-tray featuring self-curing acrylic resin (Vertex Trayplast, Vertex Dental, The Netherlands) was fabricated; the resin was blended and shaped into a dough state before curing. The final trays underwent refinement through grinding with a red acrylic bur and polishing with a white stone bur. An addition silicone (Monophase, Take 1 Advanced, Kerr, United States) was then applied to the custom tray for the impression. The tray was positioned over the artificial jaw, adhering to the manufacturer's specified working time for complete polymerization prior to removal. After tray removal, implant analogs (DANSE, SuperLine, Dentium) were secured to the impression copings. Light body wash silicone (Take 1 Advanced, Kerr, United States) was poured around the impression copings within the fitting surface until polymerization was complete as a soft tissue replica, followed by the pouring of gypsum (Type V) to produce a master model. The same custom tray was utilized repeatedly 10 times to yield a total of 10 master casts. Then, the scan bodies were seated onto the implant analogs and scanned with the laboratory scanner (KaVo ARCTICA AutoScan, Kavo Dental, Biberach, Germany), with each master cast scanned individually and exported as an STL file. Ultimately, 30 STL scans were produced, with *n* = 10 for each method.

### Alignment and Trueness Analysis

**Fig. 2:**
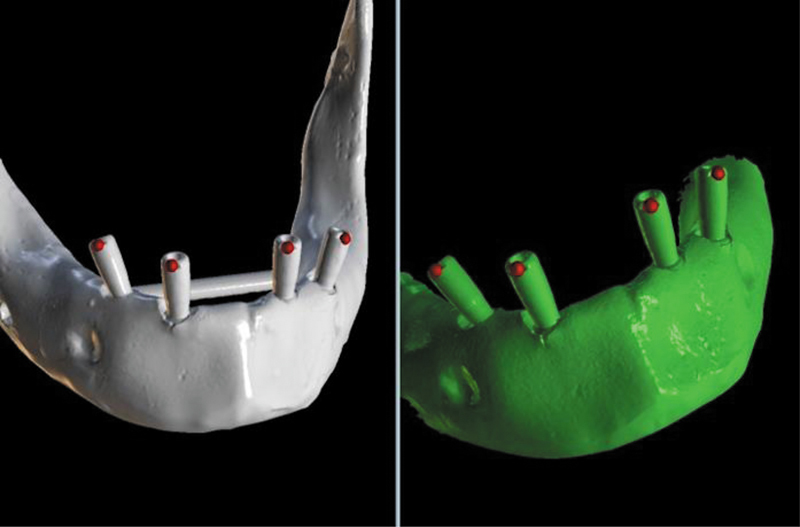
Alignment of the impression scan (right image) to the reference scan (left image) using the four defined reference points corresponding to the notch marks on each scan body.

Each scan was imported into Blue Sky Plan software (version 4.12, Blue Sky Bio, United States) for alignment with the reference scan. This alignment was based on four predefined reference points corresponding to the buccally oriented notch marks on each scan body across all implants ( Fig. 2 ). The aligned STL files were processed in Autodesk Meshmixer software (version 3.5.474, California, United States) to assess the degree of deviation between the impression scans and the reference scan. The dimensional analysis of the All-on-4 scan body zone was conducted across the *X* , *Y* , and *Z* axes for all scans and the reference using Autodesk Meshmixer. Here, the *X* -axis represented the mesiodistal dimension, the *Y* -axis reflected the apico-occlusal dimension, and the *Z* -axis delineated the buccolingual dimension. This comprehensive evaluation included a total of 90 readings, with 10 measurements per group across all dimensions for comparative statistical significance against the reference scan.

### Statistical Analysis

Normality of data distribution and variance equality were assessed through the Shapiro–Wilk's and Levene's tests, respectively ( *p* < 0.05). Statistical analysis was executed using Brown–Forsythe one-way analysis of variance (ANOVA) along with Tamhane's post hoc tests to elucidate group differences ( *p* < 0.05). All statistical procedures were conducted using IBM SPSS version 22 (SPSS Inc., Chicago, Illinois, United States).

## Results

The dataset exhibited normal distribution; however, variances were found to be unequal ( *p* < 0.05). Consequently, Brown–Forsythe one-way ANOVA coupled with Tamhane's post hoc analysis was employed for the evaluation of the data.

**Fig. 3:**
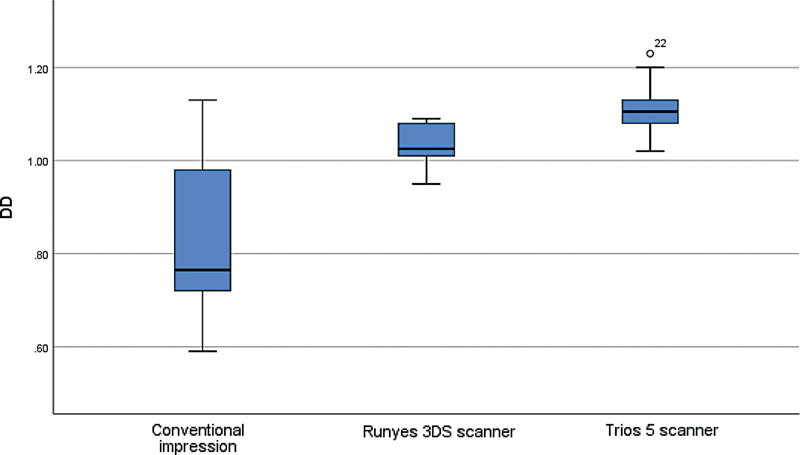
Box plot of the degree of deviation between the different impression types from the reference. Note that one outlier was spotted in the TRIOS 5 group. DD = degree of deviation.

Table 2 presents the descriptive statistics pertaining to the degree of deviation analysis. Notably, the conventional impression yielded the lowest mean deviation (0.82 mm), whereas the TRIOS 5 group exhibited the highest mean (1.11 mm). The readings from the conventional impression group demonstrated the lowest precision, as indicated by a standard deviation (SD) of ± 0.16. In contrast, the Runyes 3DS and TRIOS 5 groups displayed comparable precision (SD ± 0.05 and 0.06, respectively). Significant differences were observed among all groups, as detailed in Table 3 . Fig. 3 illustrates the box plots of the degree of deviation readings across the various groups, highlighting the wide spread of values within the conventional impression group, along with the identification of one outlier within the TRIOS 5 group.

**Table 2:** Descriptive statistics of the degree of deviation analysis mean in millimeter

Group	*N*	Mean	Standard deviation
C	10	0.82	0.16
R	10	1.02	0.05
T	10	1.11	0.06

Abbreviations: C, conventional impression; R, Runyes 3DS; T, TRIOS 5.

**Table 3:** Degree of deviation's mean analysis

( *I* ) Sample	( *J* ) Sample	Mean difference ( *I* − *J* )	Standard error	Significance
R	C	0.20	0.05	0.01 a
T	R	0.08	0.02	0.01 a
T	C	0.29	0.05	0.00 a

Abbreviations: C, conventional impression; R, Runyes 3DS; T, TRIOS 5.

a The mean difference is significant at the 0.05 level.

Descriptive statistics pertaining to the dimensional discrepancies in the scan body zone are presented in Table 4 . The reference file measurements for the scan body zone were recorded at 41.80 mm in the *X* dimension, 11.25 mm in the *Y* dimension, and 16.05 mm in the *Z* dimension. Notably, discrepancies were most pronounced along the *Z* -axis, with all three groups exhibiting greater deviations from the reference measurements in this axis compared with the others.

**Table 4:** Descriptive statistics of the abutment zone dimensional discrepancy

Axis	Impression type	*N*	Mean	Standard deviation
*X* (mesiodistally)	Control (LBS)		**41.8000**	
	C	10	41.8620	0.11679
	R	10	41.8790	0.07475
	T	10	41.9580	0.08854
*Y* (apico-occlusally)	Control (LBS)		**11.2500**	
	C	10	11.5110	0.18912
	R	10	11.2150	0.10470
	T	10	11.3240	0.11266
*Z* (buccolingually)	Control (LBS)		**16.0500**	
	C	10	15.9950	0.03719
	R	10	15.9980	0.04492
	T	10	16.1100	0.02625

Abbreviations: C, conventional impression; LBS, laboratory-based scanner; R, Runyes 3DS; T, TRIOS 5.

**Fig. 4:**
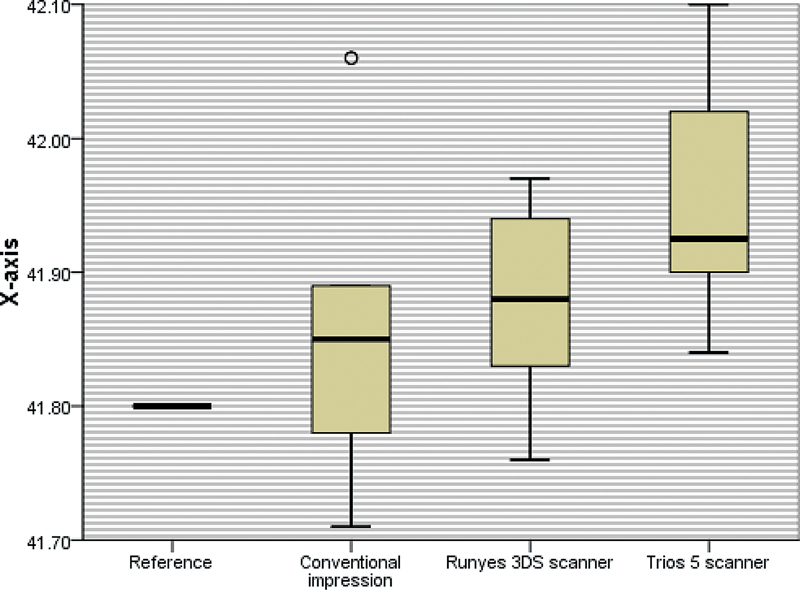
Box plot of the dimensional discrepancy in the *X* -axis (mesiodistally) between different impression methods compared with the reference. Note that one outlier was spotted in the conventional impression group.

**Fig. 5:**
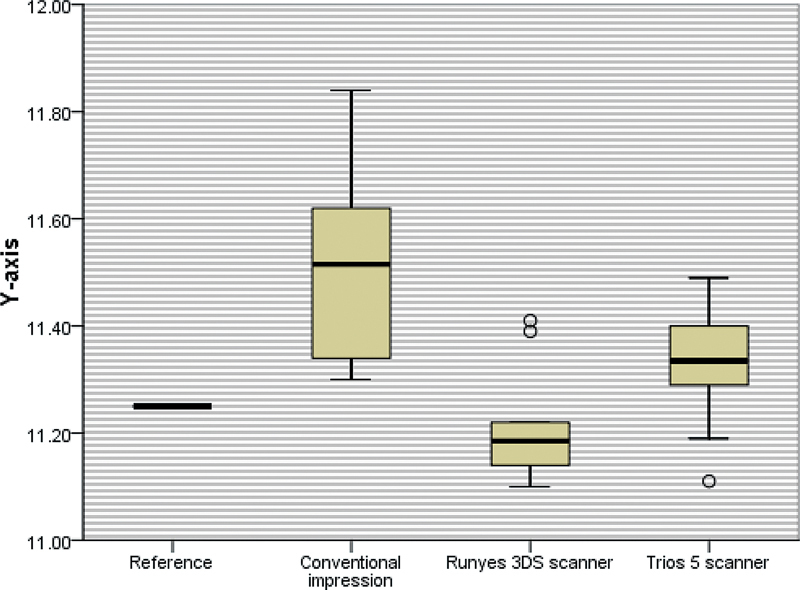
Box plot of the dimensional discrepancy in the *Y* -axis (apico-occlusally) between different impression methods compared with the reference. Note that two and one outliers were spotted in Runyes 3DS and TRIOS 5 groups, respectively.

**Fig. 6:**
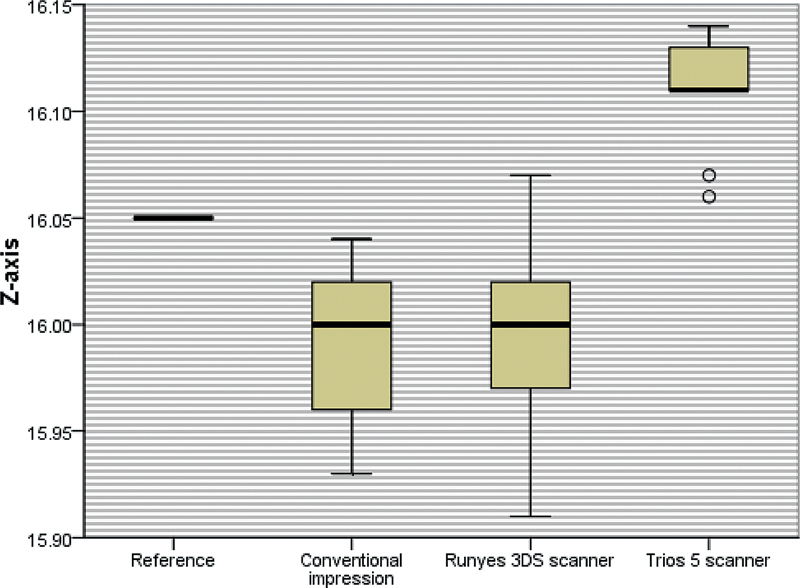
Box plot of the dimensional discrepancy in the *Z* -axis (buccolingually) between different impression methods compared with the reference. Note that two outliers were spotted in the TRIOS 5 group.

The conventional impression method demonstrated the least discrepancies in the *X* - and *Z* -axes while exhibiting the highest levels of discrepancy solely in the *Y* -axis. This method displayed the lowest precision in the *X* -axis (SD ± 0.11679) and *Y* -axis (SD ± 0.18912). Conversely, Runyes 3DS revealed the least discrepancies in the *Y* -axis and exhibited superior precision in both the *X* -axis (SD ± 0.07475) and *Y* -axis (SD ± 0.10470). In contrast, the TRIOS 5 images were characterized by the most significant discrepancies in the *X* - and *Z* -axes while maintaining the highest precision in the *Z* -axis (SD ± 0.02625) (as detailed in Table 4 ). The conventional impression method identified one outlier in the *X* -axis, whereas the Runyes 3DS and TRIOS 5 scans revealed two and one outliers in the *Y* -axis, respectively. Additionally, the TRIOS 5 scans presented two outliers in the *Z* -axis, as illustrated in Figs. 4 to 6 .

Statistical analysis indicated that the conventional impression method exhibited significant discrepancies in both the *Y* - and *Z* -axes. The TRIOS 5 scans also displayed significant discrepancies in the *X* - and *Z* -axes. Meanwhile, the Runyes 3DS readings were significantly discrepant solely in the *Z* -axis; however, the results approached significance in the *X* -axis with a borderline, *p* = 0.051 ( Table 5 ).

**Table 5:** Dimensional discrepancy's mean analysis

Dependent variable	( *I* ) sample	( *J* ) sample	Mean difference ( *I* − *J* )	Standard error	Significance
*X* (mesiodistally)	Control (LBS)	C	−0.06200	0.03693	0.559
		R	−0.07900	0.02364	0.051
		T	−0.15800 ^a^	0.02800	0.002 ^a^
*Y* (apico-occlusally)	Control (LBS)	C	−0.26100 ^a^	0.05980	0.011 ^a^
		R	0.03500	0.03311	0.899
		T	−0.07400	0.03563	0.343
*Z* (buccolingually)	Control (LBS)	C	0.05500 ^a^	0.01176	0.007 ^a^
		R	0.05200 ^a^	0.01420	0.031 ^a^
		T	−0.06000 ^a^	0.00830	0.000 ^a^

Abbreviations: C, conventional impression; LBS, laboratory-based scanner; R, Runyes 3DS; T, TRIOS 5. ^a^ The mean difference is significant at the 0.05 level.

## Discussion

The present study highlights statistically significant differences in dimensional discrepancies and deviations in scan body positions for All-on-4 implants across various impression methods, which ultimately led to the rejection of both null hypotheses. It appears that the performance of the conventional impression group is comparatively aligned with that of the Runyes 3DS group, while the TRIOS 5 group yielded less favorable results. While the conventional impression method demonstrated the best trueness (lowest mean deviation), it also exhibited the highest variability (SD ± 0.16 mm). This lower precision is likely attributable to the numerous manual steps involved, including the pouring of gypsum and the potential for analog positioning error. In contrast, the scan bodies utilized in the TRIOS 5 and Runyes 3DS groups were firmly secured in place during the scanning process, which could account for the more consistent results observed in these digital impression groups. It is noteworthy that the conventional impression method recorded the lowest mean degree of deviation (0.82 mm), and significant statistical differences were identified when comparing the digital methods to the conventional group, thereby supporting the notion that the conventional method remains a highly reliable option in this context. Interestingly, the greatest dimensional discrepancies for the conventional impression group were noted along the *Y* -axis (apico-oclusally), which may be attributable to the frequent insertion and removal of impression posts. Cumulatively, the dimensional discrepancy analysis revealed that the Runyes 3DS group exhibited the least discrepancy from the reference, followed by the conventional impression and TRIOS 5 groups, respectively. These findings present a clinical trade-off: the conventional method may, on average, get closer to the true position but with less predictability, while digital methods offer very consistent results that may be consistently offset by a certain error.

The clinical relevance of the observed deviations must be considered. The mean deviations reported (0.82–1.11 mm) exceed the widely cited threshold of 150 µm considered acceptable for a passive prosthetic fit. 20 21 This suggests that for All-on-4 cases, a single, nonsplinted digital impression without verification may carry a risk of clinically significant misfit. This underscores the necessity of employing clinical strategies to improve accuracy, such as using verification jigs or splinting techniques, regardless of the impression method chosen.

A critical consideration in this study is the selection of the LBS as the reference model. While industrial-grade coordinate measuring machines offer superior nominal accuracy, the use of a high-precision LBS is a well-established methodology in dental literature for *in vitro* comparisons of IOSs. 11 23 27 It provides a clinically relevant benchmark, as it represents the real-world gold standard to which IOS data are compared in dental laboratories. However, it is acknowledged that the stated accuracy of the LBS (20 µm) is in the same order of magnitude as the precision claimed by some IOSs. This means that the absolute trueness values reported (e.g., deviations of 0.8–1.1 mm) should be interpreted with caution, as they may include a component of error from the reference itself. Nevertheless, the relative comparison between the test groups remains valid, as all were compared against the same reference under identical conditions.

Recent studies examining the accuracy of various impression methods for complete-arch implant rehabilitation offer valuable insights that enhance the understanding of the effectiveness and limitations of these methodologies. The investigation by Zingari et al 28 provides significant comparisons among photogrammetry, IOS, and conventional impressions. Utilizing a standardized maxillary polymer resin model, their research indicates that photogrammetry achieves the highest levels of trueness and precision. This finding highlights the promising potential of photogrammetry for facilitating superior accuracy, likely due to its advanced digitization process. Conversely, the IOS method demonstrated lower accuracy, prompting important considerations regarding the reliability of digital impressions, especially in complex implant cases requiring high precision. In a complementary study, Sallorenzo and Gómez-Polo 29 compared the implant-specific PiC camera with the conventional TRIOS 3 scanner, revealing that the PiC camera provided significantly greater trueness and precision. Moreover, the research by Kaya and Bilmenoglu 30 focused on the performance of 14 IOSs in the context of the All-on-4 treatment approach. By simulating a clinical environment, this study underscored the necessity of consistency in digital impressions across diverse devices. The study highlighted the superiority of some scanners to scan these complex scenarios. However, the study also highlighted the need for caution regarding potential data loss due to artifacts or reflections, which is crucial for selecting the most suitable scanners for complex impression-taking procedures. Lyu et al 31 contributed further by comparing intraoral scanning results with conventional impressions across various scan ranges. Their findings suggest that larger scanning areas can negatively impact accuracy, particularly in cross-arch scans. The various evaluation methods employed indicate that trueness is significantly affected not only by the impression method but also by the specific clinical context, emphasizing the importance of careful consideration in choosing scanning ranges. The study conducted by Vieira et al 32 focused on the accuracy of digital intraoral versus conventional elastomeric impressions for fabricating complete implant-supported bars on a four-implant master model. Five zirconia bars were produced for each impression type using CAD/CAM technology, with vertical misfit measured in micrometers through a scanning electron microscope. The statistical analysis revealed no significant difference in vertical misfit between the two methods, regardless of screw configuration. Nonetheless, the study's scope was limited by its *in vitro* setting and focus on specific materials, which suggests the need for further exploration of additional outcome measures.

Collectively, these findings reflect a positive trend in the advancement of digital impression technology for full-arch implant rehabilitation. However, the accuracy of these methods can vary significantly based on technique, equipment, and clinical scenarios. Future research should aim to bridge the gap between *in vitro* studies and clinical applications to ensure the adoption of the most accurate and reliable impression methods, ultimately enhancing patient outcomes.

The current study's *in vitro* design presents certain limitations that may impact its applicability to clinical practice. First, the controlled environment does not fully capture the complexities present in oral conditions, where factors such as mouth opening ability, mucosal mobility, saliva, and gingival crevicular fluid significantly influence the accuracy of digital scans. Second, the comparison was made using the 3D analysis software, which is an acceptable method of analysis. However, it is more meaningful to evaluate the difference between the actual prosthesis made from these impressions to test the seating and passive fit directly. Moreover, the manual tightening of scan bodies can introduce variability; any unintentional micromovement during the impression process may result in deviations between reference and conventional impressions. In contrast, the IOSs maintained a constant configuration of both scan bodies and jaw structures between reference and digital scans. Additionally, the IOS scanned the same jaw multiple times, whereas each conventional impression corresponds to a unique master model. These considerations may complicate the interpretation of the results and highlight the importance of conducting further investigations into these variables.

## Conclusion

Within the limitations of this *in vitro* study, the following conclusions can be drawn:

1. The impression method had a statistically significant impact on the trueness and precision of scan body position capture in an All-on-4 mandibular model.
2. The conventional open-tray impression technique demonstrated the highest trueness (lowest mean deviation from the reference) but the lowest precision (highest variability).
3. The Runyes 3DS IOS demonstrated the highest precision (lowest variability), whereas the TRIOS 5 IOS exhibited the lowest trueness (greatest mean deviation from the reference).
4. Dimensional discrepancies were most pronounced along the *Z* -axis (buccolingual) for all groups.

The observed deviations highlight the inherent challenges in capturing full-arch implant positions. The superior precision of digital impressions suggests they are highly reproducible, but the overall trueness results indicate that the direct application of a single digital scan for complex All-on-4 prostheses may require validation. Future research should prioritize *in vivo* studies that correlate these dimensional measurements with the actual clinical fit of the final prosthesis to determine the clinical significance of these differences.
